# Supplementary material for: Dominance vs epistasis: the biophysical origins and plasticity of genetic interactions within and between alleles
Source: Nat Commun. 2023 Sep 9;14:5551. doi: 10.1038/s41467-023-41188-8 (PMC10492795; doi:10.1038/s41467-023-41188-8)
Supplement: Supplementary file 1 — Supplementary Information [file 41467_2023_41188_MOESM1_ESM.pdf]

Supplementary Information (SI.pdf) is provided with the manuscript.
The file contains 15 display items:
Supplementary Figures 1 – 11 and Supplementary Tables 1 – 4.

Supplementary Figure 1. Comparison of dominance (two homozygous combinations) and between-allele interaction (two heterozygous combinations) matrices.

Supplementary Figure 2. Protein folding generates intra-molecular epistasis but not dominance.

Supplementary Figure 3. Experimental validation of protein folding-only system (Model 1).

Supplementary Figure 4. Mapping mutation biophysical parameter combinations with double mutant phenotypes with the three-state model (Model 2).

Supplementary Figure 5. Interaction comparisons at altered ligand concentrations.

Supplementary Figure 6. The proportion of protein states while altering Allele 1 energy at differing ligand concentrations.

Supplementary Figure 7. Nonlinear concentration-phenotype functions differentially transform mutation interactions for folding mutants.

Supplementary Figure 8. Parameter sensitivity for interaction patterns with nonlinear concentration-phenotype functions.

Supplementary Figure 9. Scheme for making 'diploid' plasmids.

Supplementary Figure 10. Comparisons of the mutational effects between the published data and our 'diploid' heterozygote system.

Supplementary Figure 11. Flow cytometry for individual genotypes.

Supplementary Table 1. Single mutant sequences of HTH.

Supplementary Table 2. Double mutation combinations (nt).

Supplementary Table 3. PCR primers.

Supplementary Table 4. Summarised flow cytometry data.

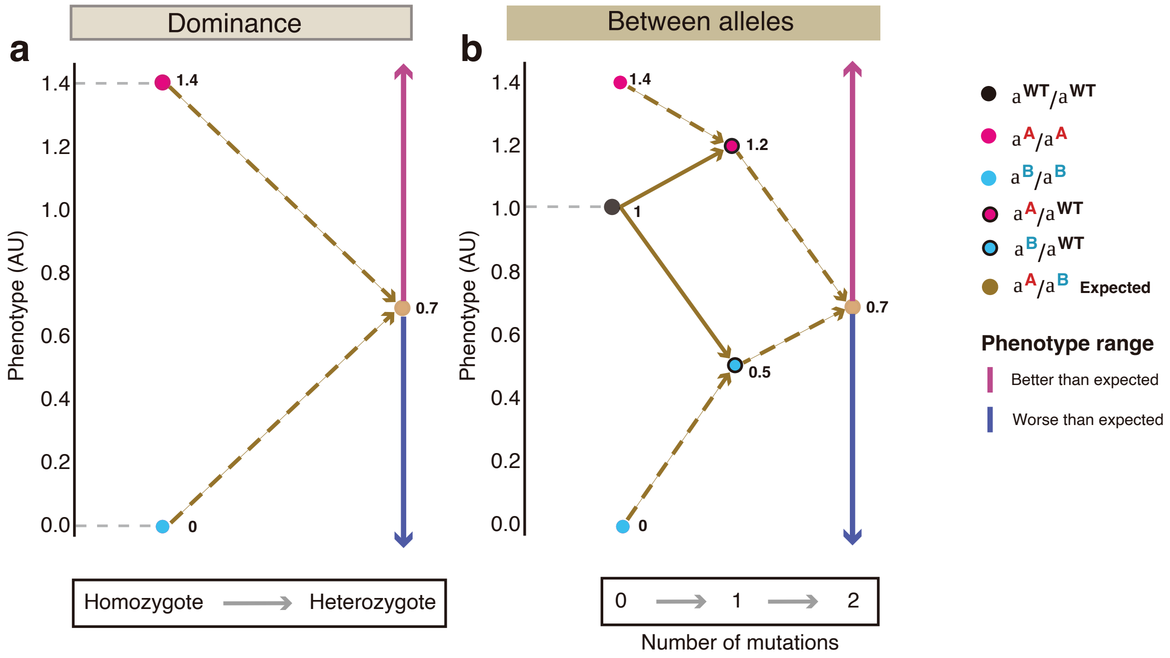

Supplementary Figure 1. Comparison of dominance (two homozygous combinations) and between-allele interaction (two heterozygous combinations) matrices. (a, b) The scheme of quantifying how two homozygotes combine (a) and how two corresponding heterozygotes combine (b) between alleles with the additive assumption. A and B indicate two different mutations of the same gene, with the forward slash separating two alleles of the same gene.

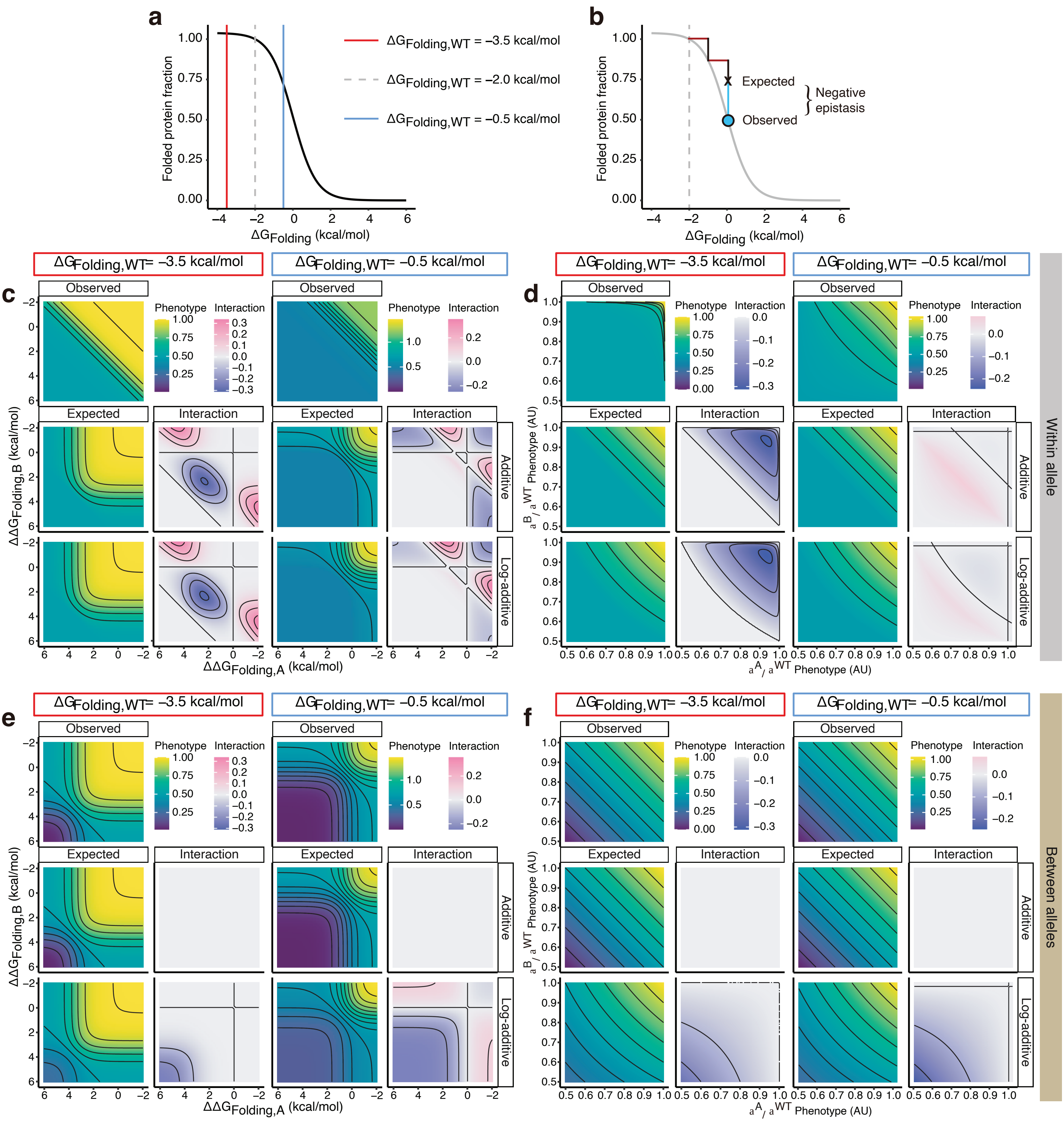

Supplementary Figure 2. Protein folding generates intra-molecular epistasis but not dominance. (a) Relationship between the free energy of protein folding and folded protein fraction. Vertical lines indicate three different settings of the wild-type stability with the dashed line showing the default setting presented in Figure 2. (b) Non-specific intra-molecular epistasis arises from the nonlinear relationship between free energy and phenotype. The grey dashed line marks the wild-type protein position. The cartoon illustrates how two destabilizing mutations increasing ΔΔG lead to negative epitasis because biophysical parameters rather than phenotypes are additive. (c-f) Heatmaps of how two mutations combine within- (c, d) or between-allele (e, f) ordered by free energy changes (c, e) or phenotypes (d, f) for a very stable protein (the red box title) or marginally stable protein (the light blue box title).

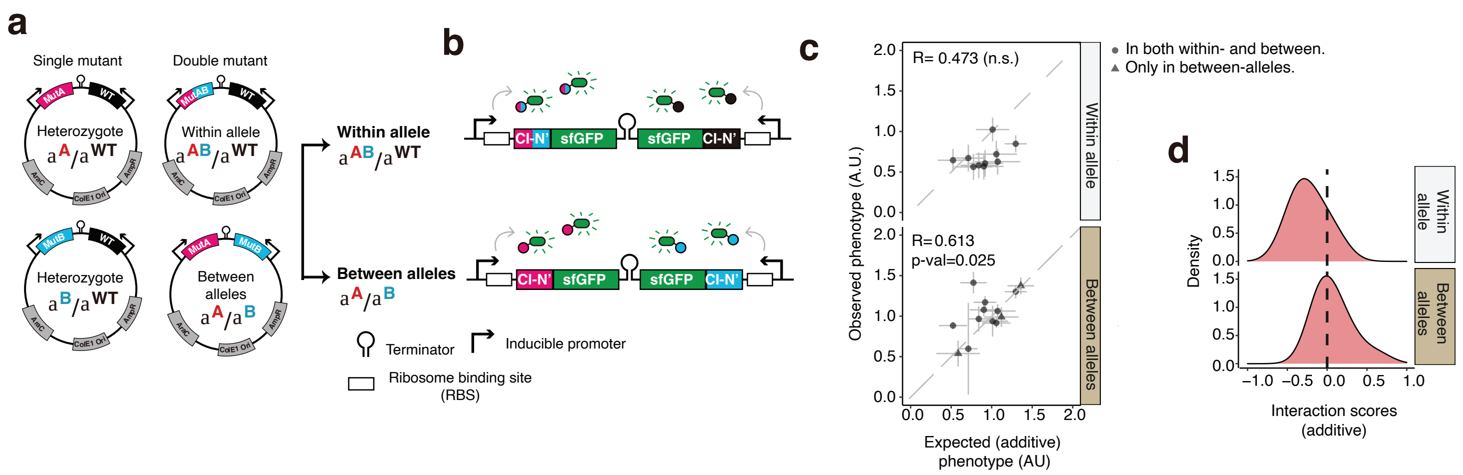

Supplementary Figure 3. Experimental validation of protein folding-only system (Model 1). (a) An overview of the plasmid designs to experimentally quantify between-allele interactions and within-allele interactions. Red and blue colours indicate two different mutations, MutA and MutB in the same gene, CI. (b) Details of the design for the folding-only model. The upper panel shows the case of within-allele mutations where one allele carries two mutations while the other allele is wild type, and the lower panel shows the between-allele case where two alleles each carry a different mutation. (c) Experimentally observed versus expected double mutant phenotypes for 10 within-allele mutation combinations and 12 between-allele combinations. Error bars denote the standard error of the mean. (d) Distribution of the interaction scores for within- and between-allele interactions using an additive expectation.

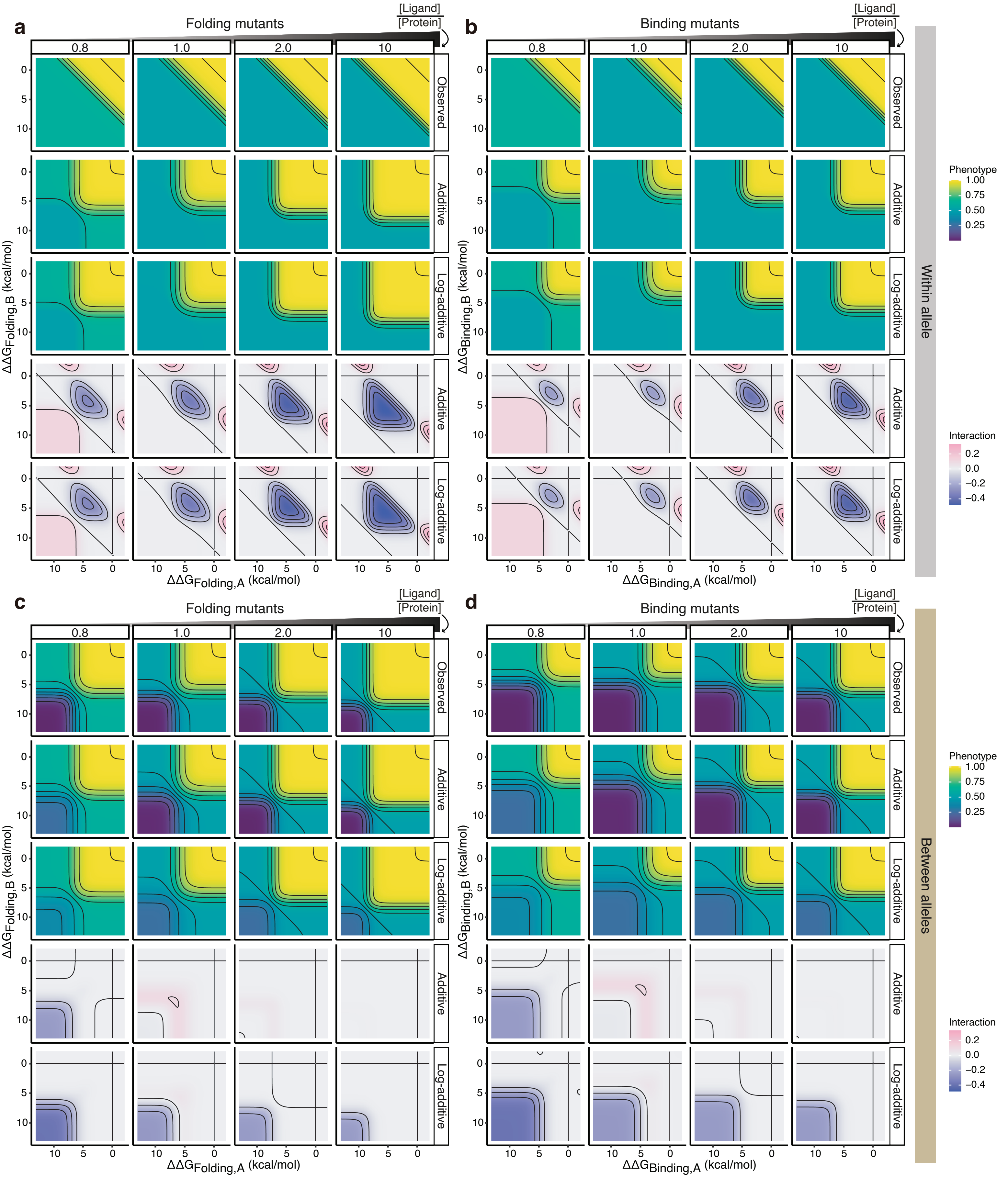

Supplementary Figure 4. Mapping mutation biophysical parameter combinations with double mutant phenotypes with the three-state model (Model 2). (a-d) Heatmaps show how two mutations combine within (a, b) or between alleles (c, d) when they both affect the same biophysical parameters: protein-folding (a, c) or ligand-binding (b, d) at different ligand-protein ratios.

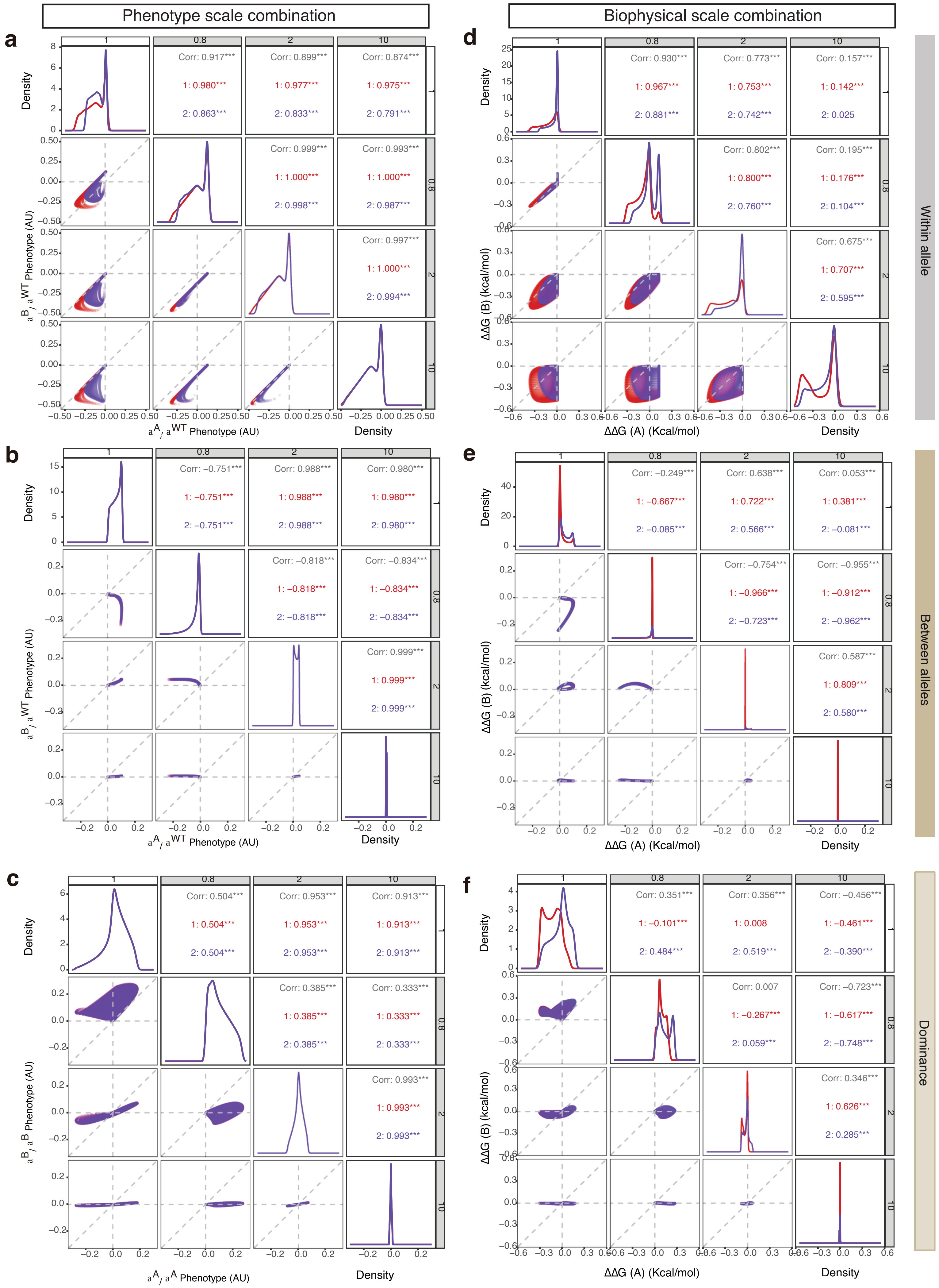

Supplementary Figure 5. Interaction comparisons at altered ligand concentrations. (a-c) Interaction scores of mutations of the same phenotype combinations for within-allele interactions (a), between-allele interactions (b) and dominance (c). (d-e) Interaction scores of mutations of the biophysical parameter combinations for within-allele interactions (d), between-allele interactions (e) and dominance (f).

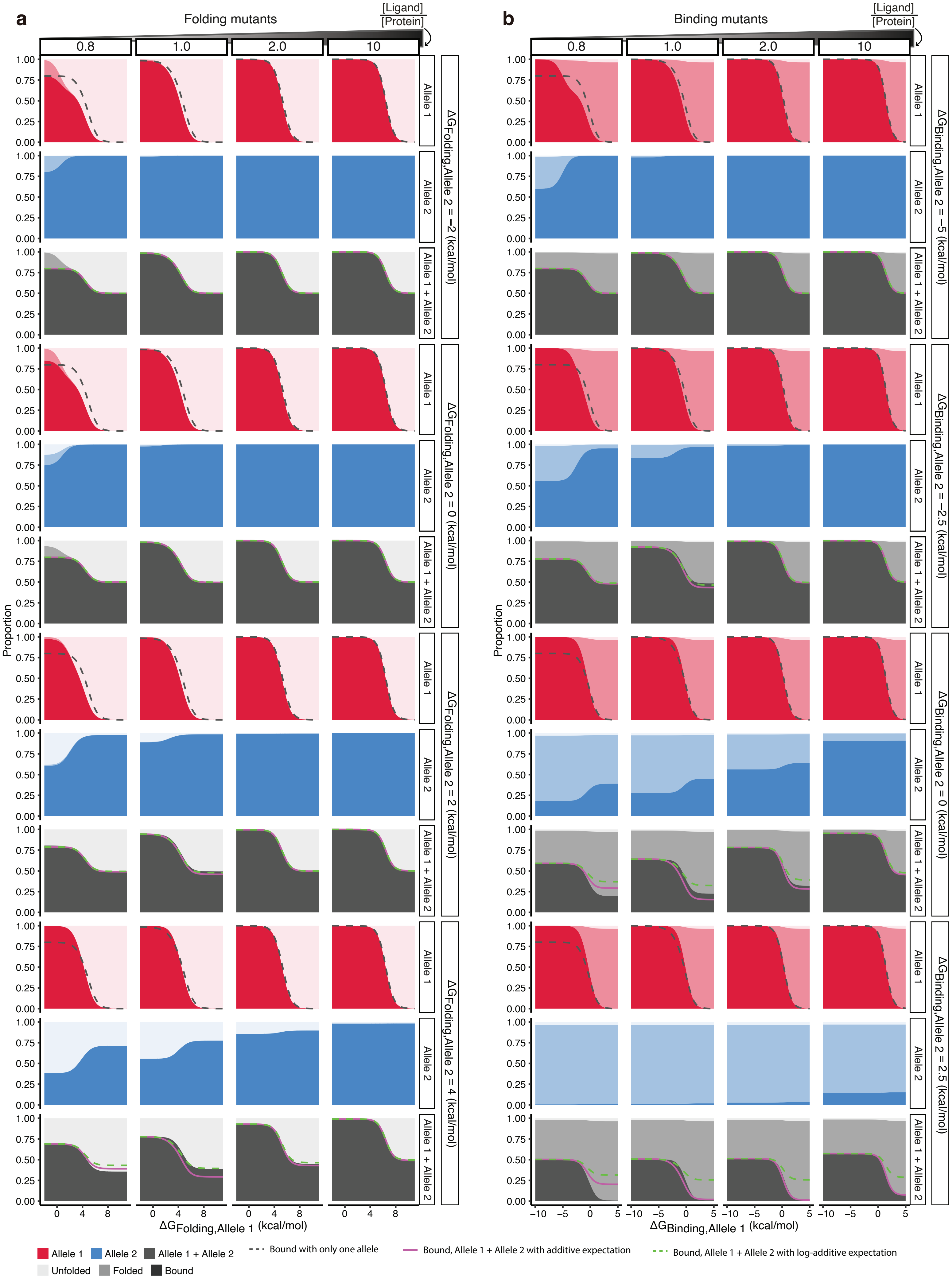

Supplementary Figure 6. The proportion of protein states while altering Allele 1 energy at differing ligand concentrations. Allele 1 and Allele 2 protein partitioning when the mutations alter Allele 1 protein folding energy (a) or binding energy to the ligand (b), based on Model 2.

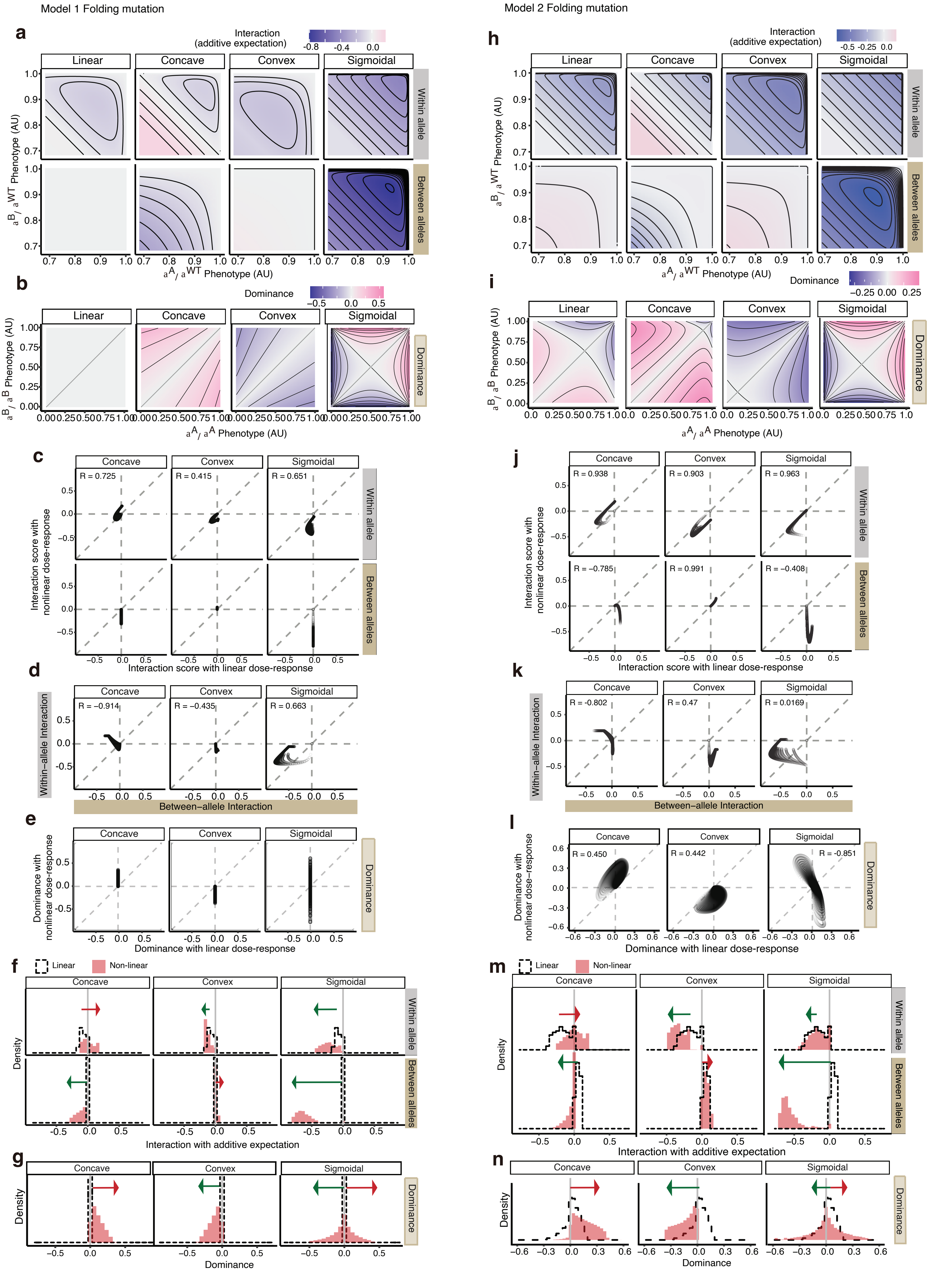

Supplementary Figure 7. Nonlinear concentration-phenotype functions differentially transform mutation interactions for folding mutants. (a-d) Interaction scores of double mutants for Model 1, for within- (the two top rows) or between alleles (the bottom row) (a) and dominance (b) with linear, concave, convex, or sigmoidal protein concentration – phenotype relationships. (c, e) Comparisons of interaction scores between double mutants within- (the two top rows) or between alleles (the bottom row) (c) and dominance (e) with or without nonlinear linking functions. (d) Comparisons of between- vs within-allele interaction scores of double mutants with nonlinear linking functions. (f, g) Interaction score distributions before and after nonlinear linking functions (f) and dominance (g). The green arrow indicates the distribution shifting towards negative values while the red arrow indicates the distribution shifting towards positive values. The arrowheads point at the range after applying the nonlinear linking functions to the phenotype. (h-n) Interaction scores of double mutants each affecting folding parameters for Model 2.

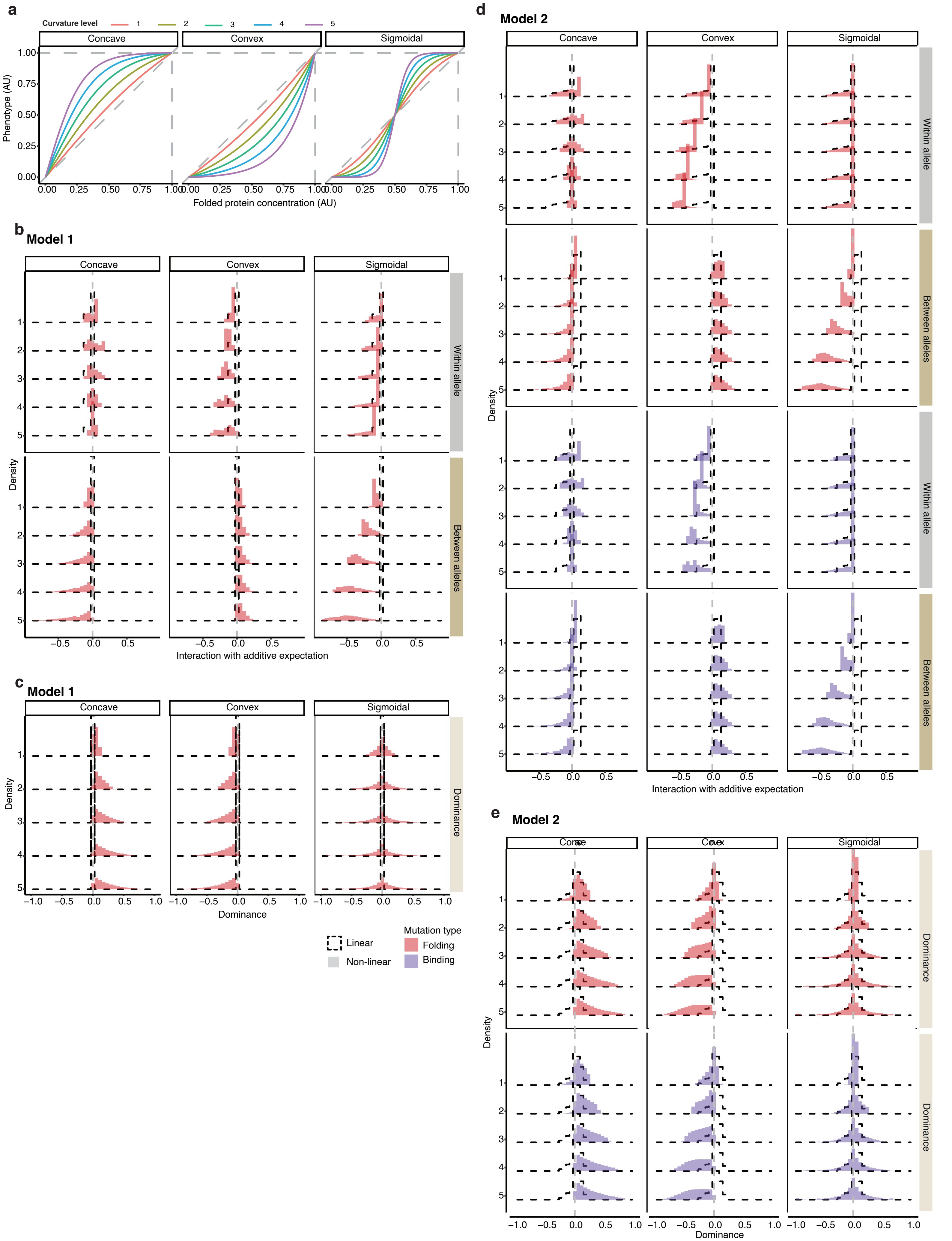

Supplementary Figure 8. Parameter sensitivity for interaction patterns with nonlinear concentration-phenotype functions. (a) Linear, concave, convex and sigmoidal linking functions for transforming protein concentrations to phenotypes. (b- e) Distribution of heterozygous mutants' interaction scores (b, d) and dominance (c, e) based on the additive expectation before and after nonlinear linking functions for Model 1 (b, c) and Model 2 (d, e), The five rows in each panel correspond to the five nonlinear curves with different curvatures in (a).

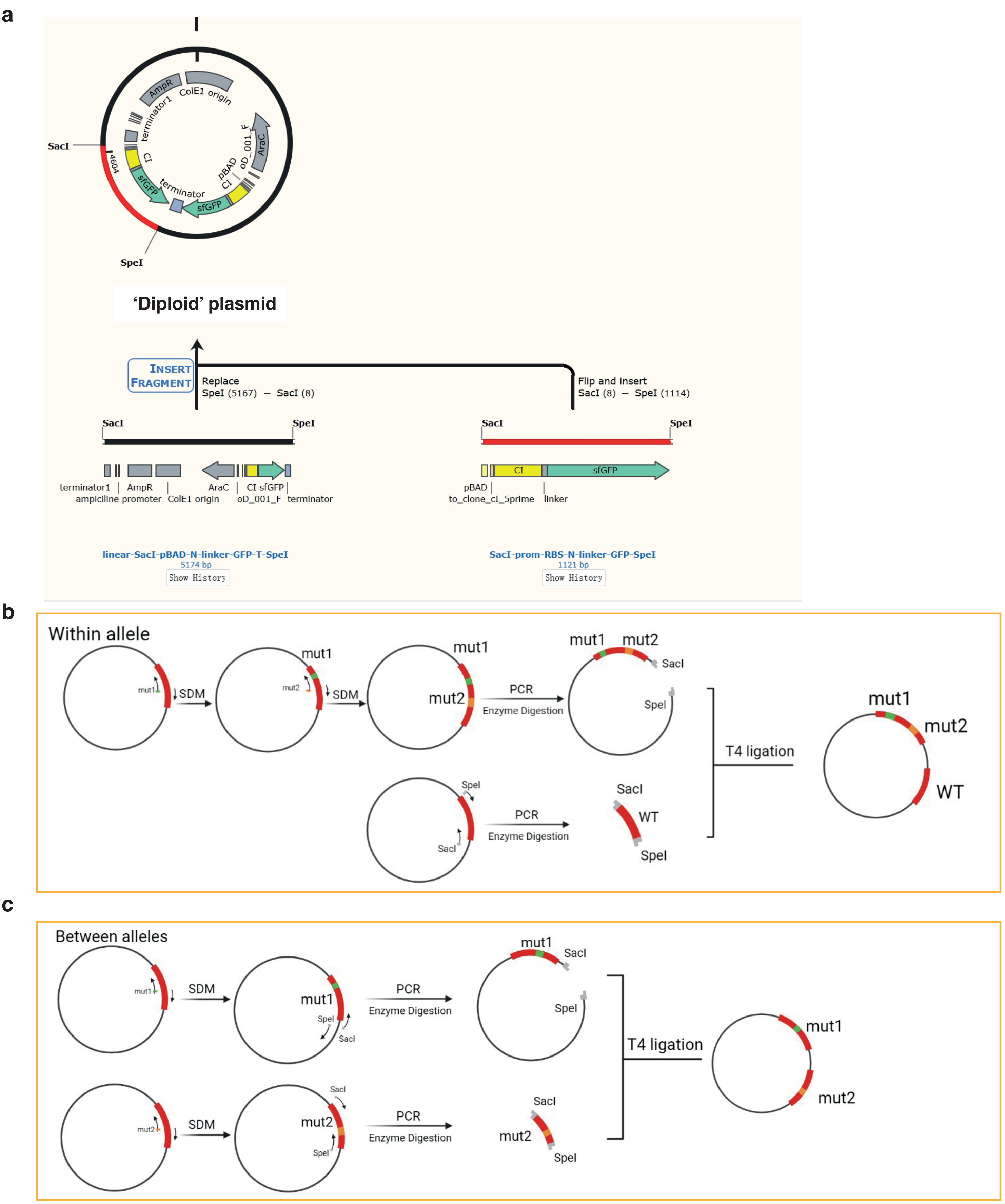

Supplementary Figure 9. Scheme for making 'diploid' plasmids. (a) Functional blocks of the wild type 'diploid' plasmids. (b, c) Scheme for making within-allele double mutations (b) and between-allele mutations (c).

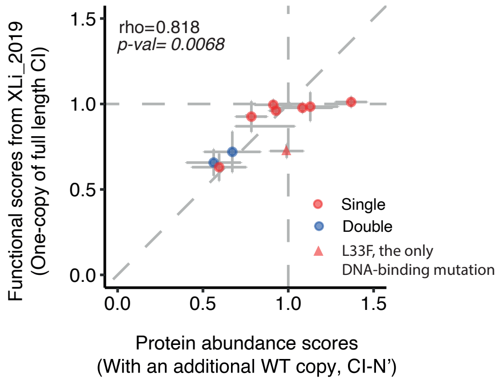

Supplementary Figure 10. Comparisons of the mutational effects between the published data and our 'diploid' hterozygote system. Error bars denote the standard error of the mean.

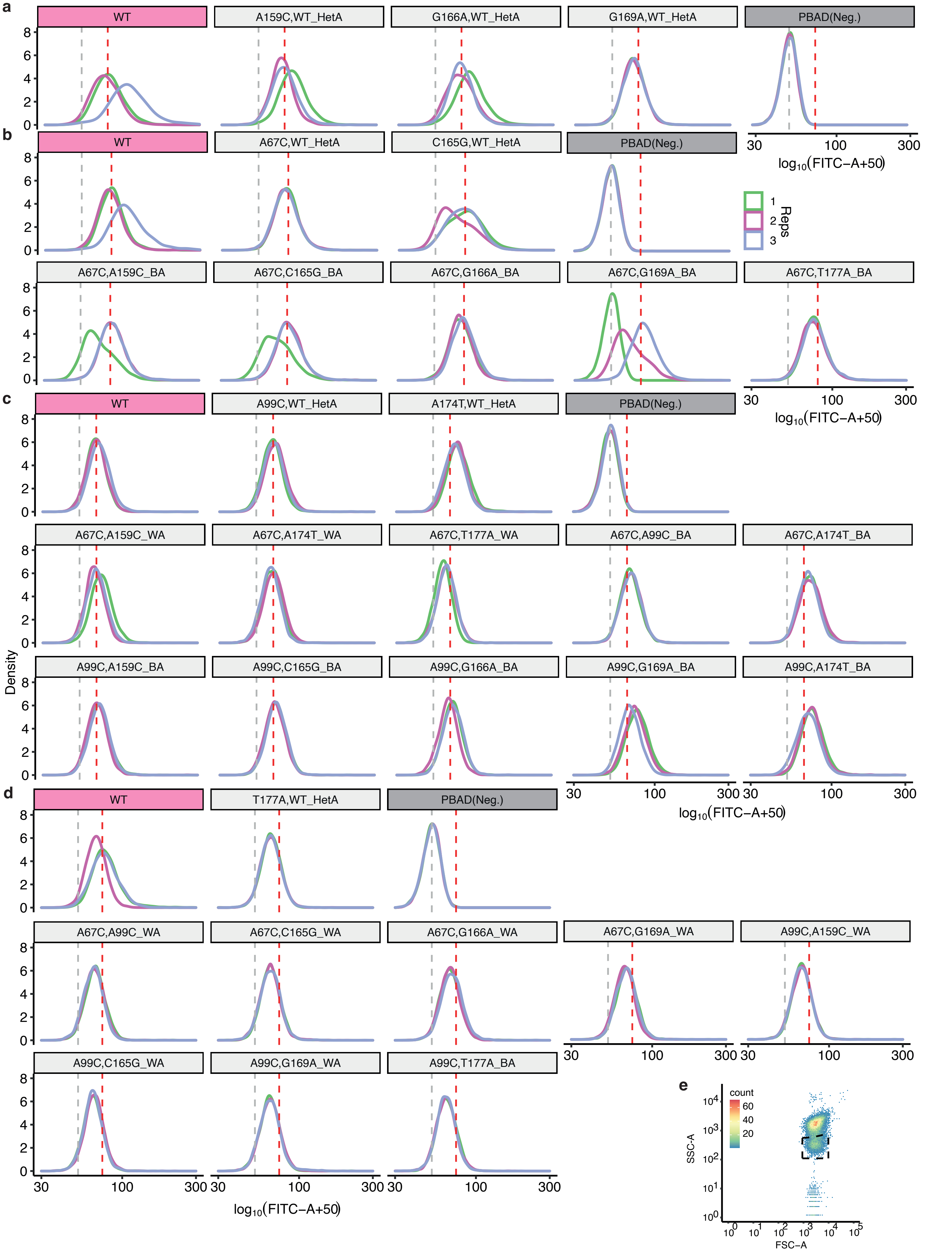

Supplementary Figure 11. FACS for individual genotypes. (a-d) Density plot of the cell FITC recordings for each genotype. Gray dashed lines mark the mean autofluorescence PBAD(Neg), and red dashed lines indicate the wild-type (WT) mean for each batch (from a - d, each annotating one batch). "HetA" indicates single mutants, "BA" indicates between-allele double mutants, and "WA" indicates within-allele double mutants. (e) A typical SSC-A and FSC-A gating of the cell population for the analysis.
