# Supplementary material for: Dominance vs epistasis: the biophysical origins and plasticity of genetic interactions within and between alleles
Source: Nat Commun. 2023 Sep 9;14:5551. doi: 10.1038/s41467-023-41188-8 (PMC10492795; doi:10.1038/s41467-023-41188-8)
Supplement: Supplementary file 3 — Reporting Summary [file 41467_2023_41188_MOESM3_ESM.pdf]

nature portfolio

Corresponding author(s): Ben Lehner, Xianghua Li

Last updated by author(s): Aug 10, 2023

Reporting Summary

Nature Portfolio wishes to improve the reproducibility of the work that we publish. This form provides structure for consistency and transparency in reporting. For further information on Nature Portfolio policies, see our Editorial Policies and the Editorial Policy Checklist.

Statistics

For all statistical analyses, confirm that the following items are present in the figure legend, table legend, main text, or Methods section.

n/a	Confirmed	
☐	☒	The exact sample size (*n*) for each experimental group/condition, given as a discrete number and unit of measurement
☒	☐	A statement on whether measurements were taken from distinct samples or whether the same sample was measured repeatedly
☒	☐	The statistical test(s) used AND whether they are one- or two-sided<br>*Only common tests should be described solely by name; describe more complex techniques in the Methods section.*
☒	☐	A description of all covariates tested
☒	☐	A description of any assumptions or corrections, such as tests of normality and adjustment for multiple comparisons
☐	☒	A full description of the statistical parameters including central tendency (e.g. means) or other basic estimates (e.g. regression coefficient) AND variation (e.g. standard deviation) or associated estimates of uncertainty (e.g. confidence intervals)
☒	☐	For null hypothesis testing, the test statistic (e.g. *F*, *t*, *r*) with confidence intervals, effect sizes, degrees of freedom and *P* value noted<br>*Give P values as exact values whenever suitable.*
☒	☐	For Bayesian analysis, information on the choice of priors and Markov chain Monte Carlo settings
☒	☐	For hierarchical and complex designs, identification of the appropriate level for tests and full reporting of outcomes
☒	☐	Estimates of effect sizes (e.g. Cohen's *d*, Pearson's *r*), indicating how they were calculated

Our web collection on statistics for biologists contains articles on many of the points above.

Software and code

Policy information about availability of computer code

Data collection	BD FACSChorus software version 1.1.20.2030 was used to analyze GFP signal of the cells.
Data analysis	R version 4.1.2 and FlowCore package was used for analysis. Custom code used in this study and deposited to GitHub: github.com/XLi-Lab/P1_Dominance_vs_Epistasis.

For manuscripts utilizing custom algorithms or software that are central to the research but not yet described in published literature, software must be made available to editors and reviewers. We strongly encourage code deposition in a community repository (e.g. GitHub). See the Nature Portfolio guidelines for submitting code & software for further information.

Data

Policy information about availability of data

All manuscripts must include a data availability statement. This statement should provide the following information, where applicable:

- Accession codes, unique identifiers, or web links for publicly available datasets
- A description of any restrictions on data availability
- For clinical datasets or third party data, please ensure that the statement adheres to our policy

The raw experimental data generated to support the conclusions of this study are included in the article and the supplementary information. The datasets generated analysed during the current study are also available in the GitHub repository, github.com/XLi-Lab/P1_Dominance_vs_Epistasis.

Research involving human participants, their data, or biological material

Policy information about studies with human participants or human data. See also policy information about sex, gender (identity/presentation), and sexual orientation and race, ethnicity and racism.

Reporting on sex and gender	n/a
Reporting on race, ethnicity, or other socially relevant groupings	n/a
Population characteristics	n/a
Recruitment	n/a
Ethics oversight	n/a

Note that full information on the approval of the study protocol must also be provided in the manuscript.

Field-specific reporting

Please select the one below that is the best fit for your research. If you are not sure, read the appropriate sections before making your selection.

☒ Life sciences ☐ Behavioural & social sciences ☐ Ecological, evolutionary & environmental sciences

For a reference copy of the document with all sections, see nature.com/documents/nr-reporting-summary-flat.pdf

Life sciences study design

All studies must disclose on these points even when the disclosure is negative.

Sample size	To experimentally evaluate our conclusion, we selected 31 samples - 8 single mutations together with 23 double mutations, the WT control and negative control. We reasoned that this number is sufficient to tell whether additive expectation versus double mutant phenotype has a linear relationship, based on our experience working on the same model using a fluorescence-based assay (Li et al., 2019).
Data exclusions	Double mutation combinations with the alpha-carbon distances within 12 angstrom in 3D spaces are removed from the analysis. The logic is that they are potentially contacting residues that are more likely to be energetically coupled with specific epistatic interactions.
Replication	Three biological replicates for each sample was performed and analyzed. Reproducibility is shown in the Supplementary Information file.
Randomization	Samples were grouped by replicates and no other grouping or randomization of samples were performed.
Blinding	Blinding is not relevant for this study because the genotype of each sample is not important for Flow Cytometry recording. Three biological replicates were performed on three different days and all the data were processed together in the later stage. Thus, not blinding during the Flow Cytometry experiment will not bias the reproducibility of each sample's phenotype or genetic interaction calculations during the analysis stage.

Reporting for specific materials, systems and methods

We require information from authors about some types of materials, experimental systems and methods used in many studies. Here, indicate whether each material, system or method listed is relevant to your study. If you are not sure if a list item applies to your research, read the appropriate section before selecting a response.

Materials & experimental systems

n/a	Involved in the study	
☒	☐	Antibodies
☒	☐	Eukaryotic cell lines
☒	☐	Palaeontology and archaeology
☒	☐	Animals and other organisms
☒	☐	Clinical data
☒	☐	Dual use research of concern
☒	☐	Plants

Methods

n/a	Involved in the study	
☒	☐	ChIP-seq
☐	☒	Flow cytometry
☒	☐	MRI-based neuroimaging

Flow Cytometry

Plots

Confirm that:

- [ ] The axis labels state the marker and fluorochrome used (e.g. CD4-FITC).
- [ ] The axis scales are clearly visible. Include numbers along axes only for bottom left plot of group (a 'group' is an analysis of identical markers).
- [ ] All plots are contour plots with outliers or pseudocolor plots.
- [ ] A numerical value for number of cells or percentage (with statistics) is provided.

Methodology

Sample preparation	Sample preparation steps are listed in the Methods.
Instrument	Cell fluorescence analysis was performed on BD Melody.
Software	Data collection with BD FACSChorus software version 1.1.20.2030, and the data analysis with R version 4.1.2, Flowcore package. Codes for the analysis is deposited at github.com/XLi-Lab/P1_Dominance_vs_Epistasis
Cell population abundance	At least 5000 cells that pass the gating strategy are collected for the further analysis for each genotype and each biological replicate.
Gating strategy	Relevant gating strategies were shown in the Methods.

- [x] Tick this box to confirm that a figure exemplifying the gating strategy is provided in the Supplementary Information.
